# Lightweight Power Line Defect Detection Based on Improved YOLOv8n

**DOI:** 10.3390/s26072112

**Published:** 2026-03-28

**Authors:** Yuhan Yin, Xiaoyi Liu, Kunxiao Wu, Ruilin Xu, Jianyong Zheng, Fei Mei

**Affiliations:** 1 School of Electrical Engineering, Southeast University, Nanjing 210096, China; yinyuhan@seu.edu.cn (Y.Y.); 220243168@seu.edu.cn (K.W.); 230259311@seu.edu.cn (R.X.); 2 Joint Graduate School (Suzhou), Southeast University, Suzhou 215123, China; 220255278@seu.edu.cn; 3 College of Energy and Electrical Engineering, Hohai University, Nanjing 211100, China; meifei@hhu.edu.cn

**Keywords:** improved YOLOv8n, lightweight model, power line defect detection, attention mechanism, loss function

## Abstract

To address the challenges of small targets, severe background clutter, and high deployment cost in UAV-based power-line defect detection, this paper proposes a lightweight defect detection model based on an improved YOLOv8n. In the downsampling stage, we design an improved lightweight adaptive downsampling module (ADownPro) to replace part of conventional convolutions, which uses a dual-branch parallel structure for stronger feature interaction and depthwise separable convolutions (DSConv) for complexity reduction. In the feature extraction stage, an integration of cross-stage partial connections and partial convolution (CSPPC) is proposed to replace the C2F module for efficient multi-scale feature fusion. In the detection head, mixed local channel attention (MLCA), which combines channel-spatial information and local–global contextual features, is introduced to strengthen defect-focused representations under complex backgrounds. For the loss function, a scale-annealed mixed-quality EIoU loss (SAMQ-EIoU) is proposed by combining iso-center scale transformation, scale factor annealing and focal-style quality reweighting to improve localization accuracy at high IoU thresholds. Experiments on a constructed dataset covering six typical defect categories show that the improved YOLOv8n achieves 91.4% mAP@0.50 and 64.5% mAP@0.50:0.95, with only 1.59 M parameters and 4.9 GFLOPs. Compared with mainstream detectors, the proposed model achieves a better balance between detection accuracy and lightweight design. In particular, compared with the recently proposed YOLOv8n-DSN and IDD-YOLO, it improves mAP@0.50 by 0.6% and 0.8%, and mAP@0.50:0.95 by 1.2% and 4.8%, respectively, while further reducing the parameter count by 1.00 M and 1.26 M, and the FLOPs by 1.7 G and 0.2 G. Moreover, the cross-dataset evaluation on the public UPID and SFID datasets further demonstrate the robustness and generalization ability of the proposed method.

## 1. Introduction

Overhead power lines are critical components of power systems, and their condition directly affects grid security and stability [1]. Defects in key components such as insulators, dampers, and grading rings may trigger protective trips, short circuits or other serious incidents [2]. Traditional manual inspection suffers from limited coverage and low efficiency [3] and thus falls short of meeting the growing demand for refined operation and maintenance. In recent years, artificial intelligence (AI) [4], especially the object detection methods based on deep learning, has received extensive attention in the field of intelligent inspection of power lines. Compared with traditional manual inspection, AI methods can automatically extract defect features from aerial images taken by drones and achieve rapid identification, thereby improving the efficiency and accuracy of inspection.

Accurate power line defect detection from UAV-acquired images faces multiple challenges. On the one hand, defect types are diverse and most defects are small-scale. As a result, details are easily lost during downsampling [5], which leads to missed detections and false alarms. On the other hand, influenced by complex backgrounds such as forests, sky, and towers, defect regions often exhibit low contrast and indistinct boundaries [6], which hinders consistent model attention to critical regions.

Moreover, although mainstream detection algorithms, including R-CNN and the YOLO series, have achieved progress in detection accuracy, their model sizes remain relatively large. According to publicly available official results, Faster R-CNN ResNet50-FPN has approximately 42 M parameters and 134 GFLOPs. Even relatively lightweight models such as YOLOv8n and YOLO11n still require about 3.2 M/8.7 GFLOPs and 2.6 M/6.5 GFLOPs in terms of parameter count and computational cost, respectively [7,8,9,10,11,12]. Meanwhile, common UAV onboard edge platforms such as VOXL 2 and Jetson Xavier NX provide public AI computing capabilities of 15+ TOPS and 21 TOPS, respectively, constrained by power consumption, weight and storage resources. Therefore, deploying existing detection models on resource-constrained edge platforms still faces considerable challenges in terms of computational scale [13].

To tackle these challenges, many researchers have proposed different optimization strategies. To improve small-object detection accuracy, Yao et al. [14] designed a C2F-DM module which combined local and global information based on YOLOv8 and proposed a BGFPN feature pyramid network with dual-layer routing attention. However, its parameter count increased to 28M, leading to elevated model complexity and limited suitability for real-time edge deployment. Lou et al. [15] proposed an MDC downsampling module to better preserve contextual information and improved YOLOv8′s feature fusion network. On the VisDrone dataset (smalls-size objects), their model improved mAP, precision, and recall by 2.5%, 1.9% and 2.1%, respectively, while the overall improvement remains limited on the PASCAL VOC2007 dataset (normal-size objects).

To improve model lightweightness, Hong et al. [16] replaced the backbone C2F blocks with a lightweight C2F-DSConv module and incorporated GSConv and a VOV-GSCSP structure into the neck, which reduced computational cost to 6.6 GFLOPs. However, its detection accuracy and robustness remained unsatisfactory. Gao et al. [17] reduced the parameter count to 3.92 M and computational cost to 10.9 GFLOPs by removing the medium and large scale detection layers and introducing SPDConv. Nevertheless, the mAP@0.50 and mAP@0.50:0.95 dropped to 95.0% and 51.4%, compared with 95.6% and 53.4% before SPDConv was introduced, suggesting that aggressive lightweighting may impair localization quality, especially at high IoU thresholds.

To balance detection accuracy and model lightweightness, Tang et al. [18] proposed YOLO-SS by optimizing the backbone, redesigning the loss function, and introducing an asymmetric sample-weighting strategy. It achieved accurate small-object detection with mAP@0.50 improving by 13.4% over YOLOv6L and recall reaching 0.641, while maintaining low computational cost. Despite these improvements, it was mainly designed for dense small-object scenarios, and its generalizability to natural scenes remained unclear. Lu et al. [19] built an insulator defect detector IDD-YOLO by incorporating a lightweight attention mechanism LCSA and GhostNet in the backbone to enhance feature extraction, and adopting GSConv and C3Ghost in the neck to reduce redundant parameters. Their final model achieved 66.2% mAP@0.50 and 38.5% mAP@0.50:0.95 on the ID-2024 dataset with only 2.85 M parameters and 5.1 GFLOPs, and further reached 99.4% mAP@0.50 and 87.2% mAP@0.50:0.95 on the SFID dataset. However, its dataset was mainly collected under clear-weather conditions and did not sufficiently cover other varying operating conditions, which limited generalization. Moreover, the model was still relatively large, posing challenges for deployment on edge devices.

In summary, recent representative methods still face three key issues. First, conventional detectors often have relatively large parameter count (2.85–42 M) and high computational cost (5.1–134 GFLOPs), which limit edge deployment. Second, the detection performance is limited in complex backgrounds and small-object recognition, which easily leads to false alarms and missed detections. Third, the localization accuracy still has deficiencies under a high IoU threshold. To address these issues, this paper proposes a lightweight power line defect detection algorithm based on an improved YOLOv8n. Our main contributions are as follows:

1. In the downsampling stage, an improved lightweight adaptive downsampling module, ADownPro, is proposed to replace part of the conventional convolutions. It adopts a dual-branch parallel structure to enhance feature interaction and reduce detail loss, while integrating depthwise separable convolution (DSConv) to control the parameter count and computational cost, thereby improving training and inference efficiency.
2. In the feature extraction stage, a lightweight module integrating cross-stage partial connections and partial convolution (CSPPC) is designed to replace the conventional C2F module. It enables feature flow and combination across stages while reducing redundant computation.
3. In the detection head, a lightweight mixed local channel attention (MLCA) mechanism is introduced. By combining channel and spatial information as well as local and global features, it strengthens the response to defect regions under complex backgrounds and improves detection accuracy.
4. For the loss function, a scale-annealed mixed-quality EIoU loss (SAMQ-EIoU) is proposed. It combines an iso-center scale transformation, a scale-factor annealing schedule, and a focal-style quality reweighting strategy. This enables progressive optimization from coarse to fine localization, improving localization accuracy at high IoU thresholds.

## 2. Methodology

### 2.1. Overall Network Structure

**Figure 1:**
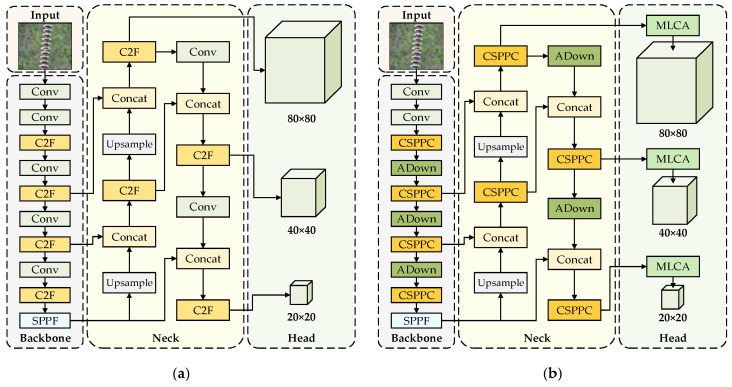
Standard and improved YOLOv8 model network structure. (**a**) Standard YOLOv8 model; (**b**) Improved YOLOv8 model.

YOLOv8 is a one-stage object detection model proposed by the Ultralytics team in 2023 [20]. It consists of three components: backbone, neck, and head. The backbone is responsible for feature extraction, the neck fuses multi-scale features from the backbone, and the head converts the fused feature maps into the final detection results. The network structure of the standard YOLOv8 is shown in Figure 1a.

The YOLOv8 family offers five configurations according to model scale and resource consumption: x, l, m, s, and n [21]. YOLOv8n is the most lightweight version. It delivers competitive detection performance with substantially fewer parameters and lower computational cost, which facilitates deployment on resource-constrained edge devices and real-time applications. In this paper, we use YOLOv8n as the baseline and its key components are further improved.

Based on this baseline, a lightweight ADownPro module is designed to reduce information loss of small-scale defects and improve efficiency during feature downsampling. A lightweight CSPPC module is introduced to replace the C2F modules to enhance multi-scale feature fusion. A lightweight attention mechanism MLCA is embedded into the detection head to strengthen the focus on defect regions under complex backgrounds. The overall network structure of improved YOLOv8 is shown in Figure 1b.

As is shown in Figure 1: (1) In the backbone and neck, the C2F modules of the standard YOLOv8 are marked in light orange, whereas the CSPPC modules of the improved YOLOv8 are marked in dark orange. (2) In the backbone and neck, the original downsampling Conv modules are marked in light green, whereas the downsampling ADownPro modules are marked in dark green. (3) In the head, the standard YOLOv8 does not include an attention mechanism, whereas the improved YOLOv8 introduces the MLCA attention module.

### 2.2. Improved Lightweight Downsampling Module: ADownPro

In the original YOLOv8n, downsampling relies on conventional stride-2 convolutions, generating feature maps at 1/8, 1/16, and 1/32 of the input resolution. However, power-line defects in UAV images are mostly small, and aggressive downsampling can easily remove the fine details required for accurate recognition. In addition, conventional convolutions introduce substantial parameters and computation, which hampers real-time deployment on resource-constrained edge devices such as UAVs.

In light of the above, this paper proposes ADownPro to replace part of the conventional convolutions, with the aim of balancing detection accuracy and model lightweightness. In ADownPro, the input feature map is initially processed by average pooling to suppress noise. Then, a dual-branch parallel structure is employed. In the first branch, max pooling is used to extract global features, followed by a 1 × 1 convolution to capture local features. In the second branch, a lightweight DSConv module [22] is adopted for feature extraction. DSConv consists of a depthwise convolution (DWConv) and a pointwise convolution (PWConv): DWConv independently extracts local spatial features within each channel, while PWConv enables cross-channel feature interaction. Finally, the outputs of the two branches are concatenated to generate the final output of the ADownPro module.

**Figure 2:**
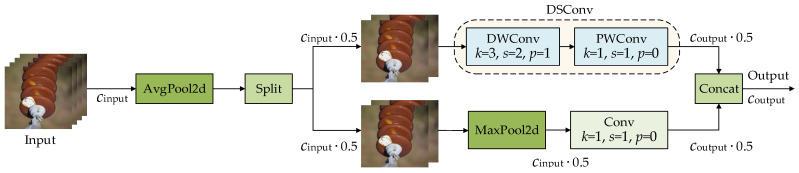
Structure of the ADownPro module.

Figure 2 shows the structure of the ADownPro module, where *C*_input_ and *C*_output_, respectively, denote the channel numbers of the input and output feature maps, *k* denotes the kernel size, *s* denotes the convolution stride, and *p* denotes the padding value.

Compared with the conventional convolution, our proposed ADownPro structure has three main advantages: (1) A dual-branch parallel design facilitates contextual feature interaction and reduces detail loss during downsampling, thereby improving the perception of small-scale power-line defects. (2) Branch 1 combines max pooling with a 1 × 1 convolution to reduce model complexity and improve training and inference efficiency. (3) Branch 2 replaces standard convolution with lightweight DSConv, which further reduces the number of parameters and computational cost while maintaining feature extraction capability.

Specifically, we compare the parameters of the original ADown module (*P*_ADown_), the proposed ADownPro module (*P*_ADownPro_) and the conventional convolution module (*P*_Conv_). Their expressions are given in Equations (1)–(3):

(1) $$P_{\mathrm{ADown}}=3^{2}\cdot \frac{c_{\mathrm{input}}}{2}\cdot \frac{c_{\mathrm{output}}}{2}+1^{2}\cdot \frac{c_{\mathrm{input}}}{2}\cdot \frac{c_{\mathrm{output}}}{2}$$

(2) $$\begin{array}{ll} P_{\mathrm{ADownPro}} & =3^{2}\cdot \frac{c_{\mathrm{input}}}{2}+1^{2}\cdot \frac{c_{\mathrm{input}}}{2}\cdot \frac{c_{\mathrm{output}}}{2}+1^{2}\cdot \frac{c_{\mathrm{input}}}{2}\cdot \frac{c_{\mathrm{output}}}{2}\\ & =\frac{9}{2}c_{\mathrm{input}}+\frac{1}{2}c_{\mathrm{input}}c_{\mathrm{output}} \end{array}$$

(3) $$P_{\mathrm{Conv}}=k^{2}\cdot c_{\mathrm{input}}\cdot c_{\mathrm{output}}$$

Further, the ratios *P*_ADown_/*P*_Conv_ and *P*_ADownPro_/*P*_Conv_ are given as follows:

(4) $$P_{\mathrm{ADown}}/P_{\mathrm{Conv}}=10/4k^{2}$$

(5) $$P_{\mathrm{ADownPro}}/P_{\mathrm{Conv}}=\frac{9}{2k^{2}c_{\mathrm{output}}}+\frac{1}{2k^{2}}$$

Conventional convolutions typically use *k* = 3. In this case, the ratio *P*_ADown_/*P*_Conv_ is 5/18, whereas *P*_ADownPro_/*P*_Conv_ is only about 1/18, indicating that ADownPro has a clear advantage in terms of lightweight design.

In summary, compared with the conventional convolution and original ADown module, ADownPro achieves better small-object detection performance with lower computational overhead. Therefore, it is adopted as the downsampling unit in this paper.

### 2.3. Lightweight Feature Extraction Module: CSPPC

In the backbone and neck of YOLOv8n, the C2F module is responsible for feature extraction and fusion. Nevertheless, its considerable computational complexity results in redundant operations and increased memory consumption, thereby posing performance bottlenecks. [23]. Therefore, a lightweight CSPPC module is designed in this paper to replace the conventional C2F module.

**Figure 3:**
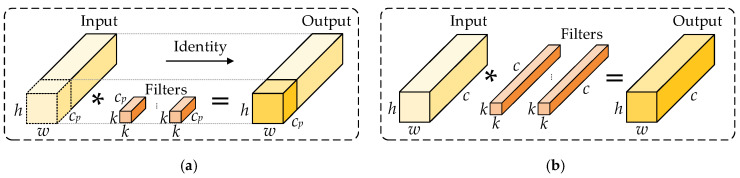
Comparison between PConv and conventional convolution. (**a**) PConv; (**b**) Conventional convolution.

The CSPPC module is built on cross-stage partial connections (CSP) and incorporates a partial convolution (PConv) strategy. CSP transmits feature information across different stages of the network, reducing redundant computation and optimizing information flow, thereby enhancing feature representation. PConv performs convolution on only part of channels in the feature map and keeps the remaining channels unchanged, which substantially lowers the parameters and computation on irrelevant regions. A comparison between PConv and conventional convolution is shown in Figure 3, where *h*, *w*, and *c* denote the height, width, and number of channels of the input feature map, respectively; *c_p_* denotes the number of channels involved in convolution (i.e., the partial channels); * denotes the convolution operation.

The computational cost of PConv (*F*_PConv_) and conventional convolution (*F*_Conv_) are given in Equations (6) and (7):

(6) $$F_{\mathrm{PConv}}=h\cdot w\cdot k^{2}\cdot c_{p}^{2}$$

(7) $$F_{\mathrm{Conv}}=h\cdot w\cdot k^{2}\cdot c^{2}$$

The ratio *F*_PConv_/*F*_Conv_ is:

(8) $$F_{\mathrm{PConv}}/F_{\mathrm{Conv}}=c_{p}^{2}/c^{2}$$

When *c_p_*/*c* is 1/4, the computational cost of PConv is only 1/16 of the computational cost of conventional convolution.

It should be noted that compared with conventional full-channel convolution, PConv reduces the channel range covered by convolution, which may weaken the feature extraction capability of some channels. However, these non-convolved channels are directly preserved through an identity path and are reintroduced into the output representation during the subsequent fusion stage. Therefore, the effect of PConv on feature representation is not simply information deletion, but rather a division of features into two parts: a convolution-enhanced branch and an original-preserved branch. The former is responsible for extracting more discriminative local spatial features, while the latter preserves the original feature information and the cross-stage feature flow.

**Figure 4:**
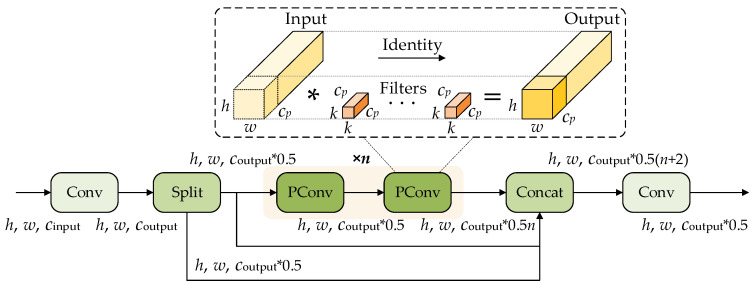
Structure of the CSPPC module.

Furthermore, the structure of the CSPPC module is shown in Figure 4, which consists of several submodules, including Conv, Split, PConv, and Concat.

As is shown in Figure 4, the input feature map first passes through a 1 × 1 convolution to perform an initial adjustment and compression of channel information, producing an intermediate feature representation. The feature map is then divided into two parts along the channel dimension by the Split module. One part is directly preserved for subsequent feature fusion, while the other part passes through two successive PConv operations to extract more discriminative local spatial features.

On the one hand, this structure preserves cross-stage feature flow through the Split–Concat mechanism, thereby reducing repeated gradients and redundant computation. On the other hand, by using PConv for efficient spatial feature extraction on partial channels, it reduces the parameter count and computational cost while still maintaining effective feature representation capability. Therefore, the CSPPC module improves network efficiency while balancing lightweight design and feature extraction capability, and enhances the robustness and detection accuracy of the model under complex backgrounds.

### 2.4. Lightweight Attention Mechanism Module: MLCA

In power line defect detection, UAV-acquired images are often affected by complex backgrounds, and defects typically appear only in local regions. Introducing an attention mechanism helps the model focus on critical areas, thereby improving defect recognition.

However, most existing attention mechanisms mainly emphasize channel information while neglecting spatial information [24], which often introduce considerable parameters and computational cost. To balance accuracy and model complexity, a lightweight MLCA module is incorporated to improve the defect detection network.

**Figure 5:**
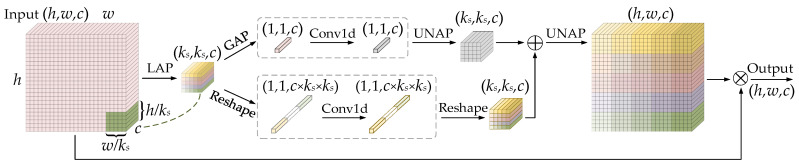
Principle of the MLCA module.

MLCA integrates channel and spatial information, as well as local and global features. With a low computational overhead, it strengthens the model’s focus on salient regions and facilitates the detection of subtle power line defects, such as insulator or damper defects. Figure 5 illustrates the principle of the MLCA module.

As shown in Figure 5, the input feature map is first converted into a *k_s_* × *k_s_* × *c* vector via local average pooling (LAP) to capture local spatial information. Next, a dual-branch structure is adopted: one branch applies global average pooling (GAP), and the other performs a reshape operation to generate a 1D vector, which are used to extract global information and local spatial information, respectively. Furthermore, features are extracted using a 1D convolution (Conv1d), and two *k_s_* × *k_s_* × *c* vectors are recovered through un-average pooling (UNAP) and reshape operations. Finally, the outputs of the two branches are fused to form hybrid attention, and UNAP is used to restore the feature map to the original input size.

According to Figure 1b, the lightweight MLCA module is integrated into the detection head and applied to the P3, P4, and P5 feature maps. This design facilitates adaptive emphasis on key regions of targets at different scales while suppressing interference from complex backgrounds, thereby improving overall accuracy and robustness.

### 2.5. SAMQ-EIoU Loss Function

The original YOLOv8n adopts CIoU as the bounding box loss, which jointly considers the overlap between the predicted and ground-truth boxes, the center distance, and the aspect-ratio difference [25]. However, the aspect-ratio term in CIoU mainly measures aspect-ratio consistency and tends to degenerate when the aspect ratios are similar. It also fails to explicitly model the absolute errors in width and height, which may limit optimization efficiency. Therefore, EIoU is adopted as the regression backbone in this paper, introducing explicit constraints on the width and height errors to improve optimization efficiency. The EIoU loss (*L_EIoU_*) is shown as follows:

(9) $$L_{EIoU}=L_{IoU}+L_{dis}+L_{asp}$$

(10) $$L_{IoU}=1-IoU$$

(11) $$L_{dis}=\frac{\rho^{2}(b^{pred},b^{gt})}{c^{2}}$$

(12) $$L_{asp}=\frac{\rho^{2}(w^{pred},w^{gt})}{c_{w}^{2}}+\frac{\rho^{2}(h^{pred},h^{gt})}{c_{h}^{2}}$$

In the equations: *IoU* denotes the intersection-over-union between the predicted and ground-truth boxes; *L_IoU_* denotes the IoU loss; *L_dis_* denotes the distance loss; *L_asp_* denotes the aspect-ratio loss; *ρ*(·) denotes the Euclidean distance; *b^pred^*, *w^pred^* and *h^pred^* denote the center coordinates, width and height of the predicted box, respectively; *b^gt^*, *w^gt^* and *h^gt^* denote the center coordinates, width and height of the ground-truth box, respectively; *c* denotes the diagonal length of the smallest enclosing rectangle covering both the predicted and ground-truth boxes; *c*_w_ and *c*_h_ denote the width and height constants, respectively.

Small defect targets and complex backgrounds in power line imagery often lead to a dominance of low-quality predicted boxes during training, which dominate gradient updates. This causes the model to focus more on “roughly placing the predicted box near the target” (coarse alignment) but struggle to “accurately fit the predicted box to the object boundaries” (fine localization), thereby limiting localization performance at high IoU thresholds. To address this issue, we propose the SAMQ-EIoU loss. By integrating an iso-center scale transformation, a scale-factor annealing schedule [26], and a focal-style quality reweighting strategy [27], it dynamically adjusts the regression contributions of predicted boxes with different qualities, gradually shifting the training focus from coarse alignment to fine localization.

First, inspired by UIoU, an iso-center scale transformation is applied to the predicted and ground-truth boxes, and a new *IoU_r_* is computed in the scaled domain, as shown in Equations (13) and (14):

(13) $$T_{r}(B)=(x,y,rw,rh)$$

(14) $$IoU_{r}=IoU\left(T_{r}(B^{pred}),T_{r}(B^{gt})\right)$$

In the equations: *T_r_*(·) denotes the scale transformation operation; *B* denotes a bounding box; *x*, *y*, *w* and *h* denote the x-coordinate of the box center, the y-coordinate of the box center, the width of the box, and the height of the box, respectively; *r* denotes the scale factor; *IoU*(·) denotes the intersection-over-union operation; *B^pred^* and *B^gt^* denote the predicted box and the ground-truth box, respectively.

Second, to enable progressive optimization from coarse alignment to fine localization, an annealing schedule is applied to the scale factor *r*. At the early stage of training, a larger *r* is used to reduce the penalty of 1 − *IoU_r_*, so that optimization focuses primarily on coarse alignment. As training proceeds, *r* is gradually decreased to mainly increase 1 − *IoU_r_* for high-quality samples, thereby strengthening fine localization. The scale factor is set as follows:

(15) $$r(t)=r_{s}+(r_{e}-r_{s})\beta (t)$$

(16) $$\beta (t)=\frac{1-\cos(\pi t/T)}{2}\in [0,1]$$

In the equations: *t* denotes the current training epoch; *r_s_* and *r_e_* denote the initial and final value of the scale factor, respectively; *β*(*t*) denotes the cosine annealing scheduling function; *T* denotes the total number of training epochs.

Third, to mitigate the imbalance in regression contributions from samples of different qualities, a focal-style quality reweighting strategy is introduced. A weighting function *w*(*t*) is used to reallocate each sample’s loss contribution as follows:

(17) $$w(t)=(1-\beta (t)){\left(1-IoU_{r}\right)}^{\gamma }+\beta (t){\left(IoU_{r}\right)}^{\gamma }$$

In the equation: *γ* is the focusing factor that controls the strength of reweighting.

For low-quality samples, *IoU_r_* is relatively small and (1-*IoU_r_*)*^γ^* is relatively large. With a small *β*(*t*) at the early stage of training, the weight *w*(*t*) is biased toward low-quality samples. This increases their loss weights and gradient contributions, driving predicted boxes to quickly converge toward the ground truth. As training proceeds, *β*(*t*) gradually increases and the weight smoothly shifts from the low-quality term to the high-quality term (*IoU_r_*)*^γ^*, so that high-quality samples with larger *IoU_r_* receive higher weights, further strengthening boundary alignment and fine localization.

Finaly, the proposed SAMQ-EIoU regression loss is given by Equation (18):

(18) $$L_{SAMQ-EIoU}=w(t)\cdot \left[(1-IoU_{r})+L_{dis}+L_{asp}\right]$$

This design adjusts the IoU loss in the scaled domain through scale-factor annealing and combines it with focal-style weighting for quality-guided gradient allocation. Consequently, it promotes fine regression of high-quality predicted boxes while maintaining training stability, thereby improving localization accuracy and robustness for power line defects at high IoU thresholds.

## 3. Case Study

### 3.1. Data Preparation and Experimental Environment

**Figure 6:**
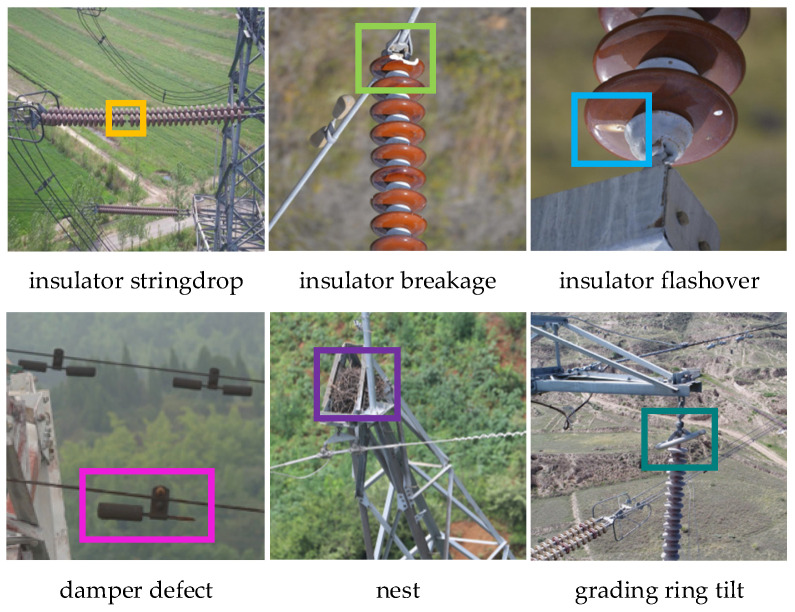
Representative samples of power line defect images.

The dataset in this study consists of two parts: one collected by our research group using UAVs, and the other obtained from public online sources. In total, the dataset contains 6050 images covering six typical defect categories: insulator stringdrop, insulator breakage, insulator flashover, damper defect, nest, and grading ring tilt. Representative samples are shown in Figure 6.

**Figure 7:**
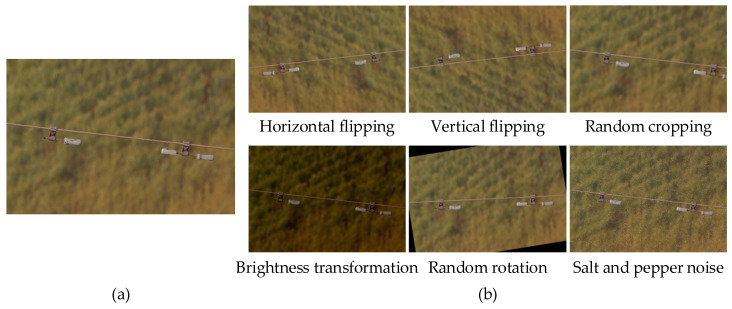
Illustration of data augmentation results. (**a**) Original image; (**b**) Data augmentation.

To mitigate class imbalance and improve model generalization, multiple data augmentation strategies are applied to categories with limited training samples, including horizontal flipping, vertical flipping, random cropping, brightness transformation, random rotation, and the addition of salt-and-pepper noise. After augmentation, the dataset contains 6165 images. Figure 7 illustrates an example of data augmentation results.

**Figure 8:**
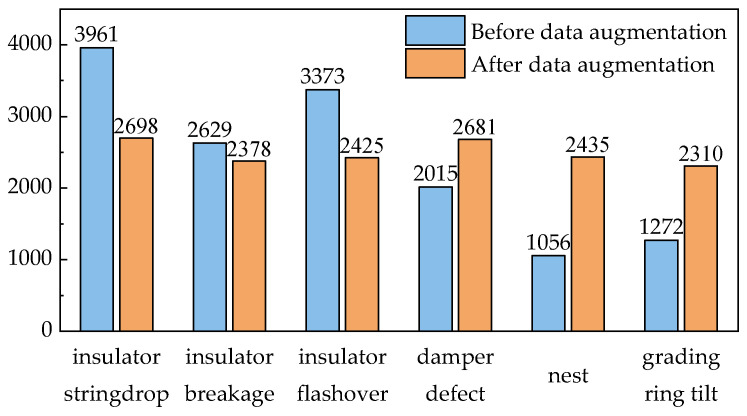
Numbers of labeled boxes for each category before and after data augmentation.

This paper uses LabelImg to annotate defect bounding boxes, and the labels are saved in TXT format. The numbers of labeled boxes for each category are shown in Figure 8.

As can be seen from Figure 8, a total of 14,306 defect instances are annotated before augmentation, and the class distribution becomes more balanced after augmentation, reaching 14,927 defect instances.

Further, the dataset is split into the training, validation, and test sets at a ratio of 8:1:1, corresponding to 4933 training images, 616 validation images, and 616 test images.

Moreover, our experimental environment and hyperparameter settings are listed in Table 1 and Table 2. All experiments are conducted under the same configuration.

### 3.2. Evaluation Metrics

To evaluate detection accuracy, this paper uses precision (P), recall (R), average precision (AP), and mean average precision (mAP) as evaluation metrics. Their definitions are given as follows:

(19) $$P=\frac{T_{P}}{T_{P}+F_{P}}$$

(20) $$R=\frac{T_{P}}{T_{P}+F_{N}}$$

(21) $$AP_{i}=\int_{0}^{1}P(R)dR$$

(22) $$mAP=\frac{1}{N}\sum_{\begin{array}{c} i=1 \end{array}}^{N}AP_{i}$$

In the equations: *T*_P_, *F*_P_ and *F*_N_ denote the numbers of true positives (predicted positive and actually positive), false positives (predicted positive but actually negative), and false negatives (predicted negative but actually positive), respectively; *AP_i_* denotes the average precision of the i-th class; *P*(*R*) denotes the precision–recall curve; *N* denotes the total number of classes in the dataset.

For the metric mAP, mAP@0.50 and mAP@0.50:0.95 are used to reflect detection performance at different levels of localization accuracy. Specifically, mAP@0.50 denotes the mAP at an IoU threshold of 0.50, while mAP@0.50:0.95 denotes the mean mAP averaged over IoU thresholds from 0.5 to 0.95 with a step size of 0.05, which provides a comprehensive assessment of fine localization performance at high IoU thresholds.

To evaluate lightweight level, this paper adopts the number of parameters (Params), floating-point operations (FLOPs) and frames per second (FPS) as metrics. Their formulations are given as follows:

(23) Params=K(cHW+1)

(24) FLOPs=nhwcKHW

(25) $$FPS=N_{\mathrm{frame}}/t_{\mathrm{total}}$$

In the equations: *n* denotes the number of input feature maps; *h*, *w* and *c* denote the height, width and number of channels of the input feature map, respectively; *K*, *H*, and *W* denote the number, height, and width of convolution kernels, respectively; *N*_frame_ denotes the total number of frames; *t*_total_ denotes the total time required for model preprocessing, inference and post-processing.

Although the increase in Params and FLOPs can enhance model capacity, it comes with higher computational cost, harder training, and greater overfitting risk.

### 3.3. Detection Results and Analysis

This section analyzes the detection results of the improved YOLOv8n on the constructed dataset and compares them with those of the original YOLOv8n. The AP@0.50 for each class and the overall mAP@0.50 and mAP@0.50:0.95 are shown in Table 3.

As can be seen from Table 3, the improved YOLOv8n increases mAP@0.50 from 88.3% to 91.4% (+3.1%) and mAP@0.50:0.95 from 56.6% to 64.5% (+7.9%). This indicates that the proposed method enhances overall defect detection, and the improvement is more substantial at higher IoU thresholds, reflecting stronger fine localization capability. At the class level, the AP@0.50 for grading ring tilt improves by 9.0%, and insulator breakage and insulator flashover improve by 3.3% and 6.7%, respectively. The remaining categories show smaller gains, and an overall stable improvement trend is maintained.

**Figure 9:**
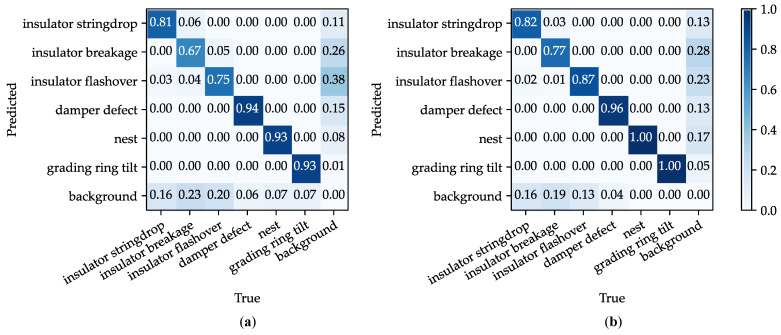
Comparison of confusion matrices between improved YOLOv8n and YOLOv8n. (**a**) YOLOv8n; (**b**) Improved YOLOv8n.

Figure 9 presents the confusion matrices for the original and improved YOLOv8n models. The results reveal higher diagonal and lower off-diagonal values for our model, suggesting improved class discrimination.

### 3.4. Ablation Experiment

#### 3.4.1. Ablation Experiment on the Proposed Module

To verify the effectiveness of each module in the improved YOLOv8n, an ablation experiment is conducted with the original YOLOv8n as the baseline. Under the same training procedure, environment and hyperparameter settings, we adopt the control variable method to sequentially add ADownPro, CSPPC, MLCA and SAMQ-EIoU loss. The contribution of each module to model performance is quantitatively evaluated. The results of the ablation experiment are reported in Table 4.

As shown in Table 4, after introducing ADownPro into the baseline YOLOv8n, the Params decreases from 3.01 M to 2.47 M, the FLOPs decreases from 8.2 G to 7.1 G, and the FPS increases from 130.3 to 136.9. Meanwhile, mAP@0.50 increases from 88.3% to 89.4%, and mAP@0.50:0.95 increases from 56.6% to 57.0%. These results indicate that ADownPro can preserve key information of small defect targets and effectively reduce the computational overhead during downsampling.

On this basis, after replacing the C2F modules with CSPPC, the Params drops substantially to 1.58 M, the FLOPs is further reduced to 4.9 G, and the FPS further increases to 157.4, while mAP@0.50 and mAP@0.50:0.95 change only slightly to 88.8% and 56.8%, respectively. This indicates that the main role of CSPPC is to reduce redundant computation through more efficient feature extraction.

After introducing MLCA, mAP@0.50 and mAP@0.50:0.95 increase to 89.8% and 59.2%, respectively, while Params and FLOPs remain nearly unchanged. The FPS decreases slightly from 157.4 to 151.8. This shows that MLCA can effectively enhance the model’s focus on key regions with almost no additional computational cost, thereby compensating for the possible loss of feature representation caused by the preceding CSPPC.

Finally, after introducing SAMQ-EIoU, mAP@0.50 increases from 89.8% to 91.4%, and mAP@0.50:0.95 increases significantly from 59.2% to 64.5%. At the same time, the Params, FLOPs and FPS remains almost unchanged. These results indicate that SAMQ-EIoU can effectively optimize the bounding-box regression process at the loss-function level, especially improving localization accuracy and bounding-box fitting quality under high IoU thresholds.

Overall, ADownPro and CSPPC mainly undertake the tasks of model lightweighting and preservation of small-target features, MLCA mainly serves feature enhancement under complex backgrounds, and SAMQ-EIoU mainly focuses on fine localization optimization under high IoU thresholds. Therefore, these four modules complement each other in three aspects, namely lightweight design, small-defect detection, and fine localization.

#### 3.4.2. Ablation Experiment on Data Augmentation

To verify the influence of different data augmentation strategies on model detection performance, we further conducted a data augmentation ablation study. Under the same network structure, training strategy, experimental environment, and hyperparameter settings, the detection performance under different augmentation combinations was compared and analyzed.

Starting from the dataset without data augmentation (None), we gradually introduced horizontal flipping (M1), vertical flipping (M2), random cropping (M3), brightness transformation (M4), random rotation (M5), and salt-and-pepper noise (M6). Under each setting, mAP@0.50 and mAP@0.50:0.95 were recorded. The results are shown in Table 5.

As shown in Table 5, compared with the setting without data augmentation, the overall detection performance of the model improved after introducing augmentation strategies. Although mAP@0.50 and mAP@0.50:0.95 showed slight fluctuations during the gradual introduction of different augmentation methods, the model achieved the best detection performance under the complete augmentation strategy. This indicates that the effects of different augmentation methods on model performance are not exactly the same, but a reasonable combination of multiple augmentation strategies can effectively improve the overall detection capability of the model. Since different augmentation methods have different working mechanisms, their combined use can improve the training sample distribution from multiple aspects, thereby leading to more stable performance gains.

### 3.5. Comparative Experiment

To validate the superiority of the proposed method, comparative experiments are conducted on the constructed power line defect dataset. The compared methods include several mainstream object detectors, namely Faster R-CNN, YOLOv5n, YOLOv8n, YOLOv8s, YOLOv10n, and YOLOv11n, as well as the recently proposed lightweight defect detection methods YOLOv8n-DSN [16] and IDD-YOLO [19].

#### 3.5.1. Comparative Experiment Results

To ensure fairness, all models were trained and evaluated under the same training procedure, environment and hyperparameter settings. The experimental environment and hyperparameter settings are given in Table 1 and Table 2 in Section 3.1, respectively. For the YOLO-series methods, except for the hyperparameters listed in Table 2, the remaining structure-related settings follow the official default configurations. For Faster R-CNN, the backbone is set to ResNet-50-FPN, the RPN fg IoU threshold is 0.7, the RPN bg IoU threshold is 0.3, the RPN batch size per image is 256, the RPN positive fraction is 0.5, the box_fg_iou_thresh is 0.5, the box_bg_iou_thresh is 0.5, the box_positive_fraction is 0.25, and the box_batch_size_per_image is 512.

The results of comparative experiments are reported in Table 6.

As shown in Table 6, in terms of detection accuracy, the improved YOLOv8n achieves the best performance among all compared methods, reaching 91.4% mAP@0.50 and 64.5% mAP@0.50:0.95. Compared with YOLOv10n and YOLOv11n, the proposed method improves mAP@0.50 by 6.0% and 3.9%, respectively, and improves mAP@0.50:0.95 by 11.3% and 9.9%, respectively. Compared with the recently proposed YOLOv8n-DSN and IDD-YOLO, the proposed method still improves mAP@0.50 by 0.6% and 0.8%, respectively, and mAP@0.50:0.95 by 1.2% and 4.8%, respectively. These results indicate that, in power-line defect detection scenarios characterized by both complex backgrounds and small targets, the proposed method has more obvious comprehensive advantages in both defect detection capability and fine localization capability.

In terms of lightweight performance, the proposed method has only 1.59 M Params and 4.9 G FLOPs, which are significantly lower than those of the other models. Compared with YOLOv8n, the Params is reduced by 1.42 M and the FLOPs are reduced by 3.3 G. Even compared with the latest lightweight methods YOLOv8n-DSN and IDD-YOLO, the proposed method still reduces the Params by 1.00 M and 1.26 M, respectively, and reduces the FLOPs by 1.7 G and 0.2 G, respectively. These results demonstrate that the proposed model is able to achieve better detection performance with lower parameter count and computational overhead. Besides, the proposed method achieves 152.1 FPS, which is the highest among all compared methods.

To conclude, the improved YOLOv8n achieves a better balance between accuracy and lightweight design, and can better meet the practical requirements of power transmission and distribution line defect detection due to inference speed.

#### 3.5.2. Robustness and Stability Analysis

**Figure 10:**
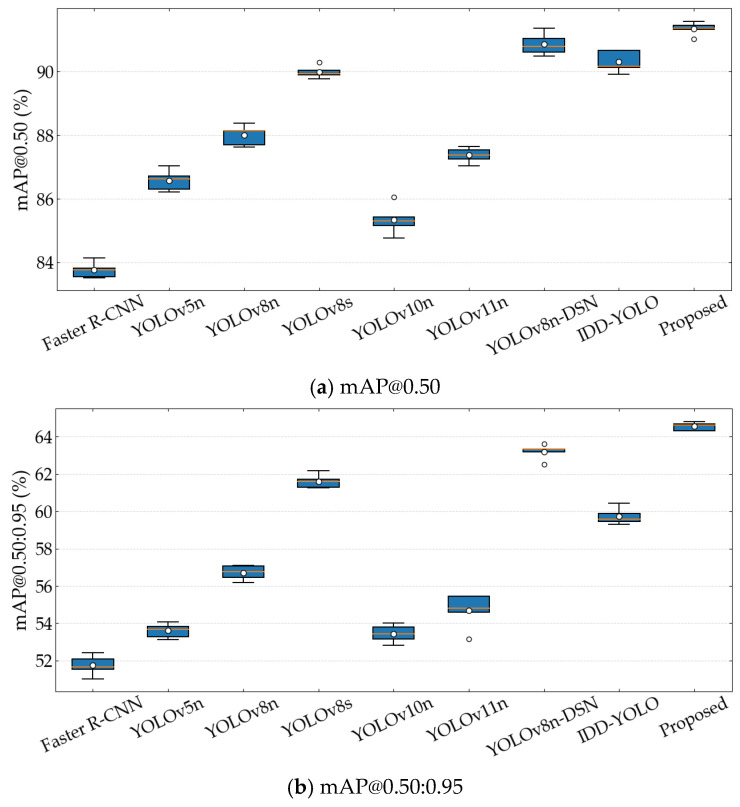
Distributions of the repeated experimental results.

To further verify the robustness and stability of the experimental results, under the same training procedure, environment, and hyperparameter settings, each compared method was trained independently five times, and the mAP@0.50 and mAP@0.50:0.95 on the validation set were recorded. The distributions of the repeated experimental results are shown in Figure 10.

As shown in Figure 10, the proposed method achieves the highest median values for both mAP@0.50 and mAP@0.50:0.95, while also showing a relatively small variation range. The standard deviations over the five runs are only 0.20 and 0.22, respectively. These results indicate that the proposed method not only achieves better detection accuracy, but also shows good stability across multiple independent training runs. In contrast, although some compared models can obtain relatively high metrics in a single run, their median values are clearly lower than those of the proposed method. This further verifies the overall advantage of the proposed method in both detection performance and result robustness.

#### 3.5.3. Convergence Curves and Detection Results

**Figure 11:**
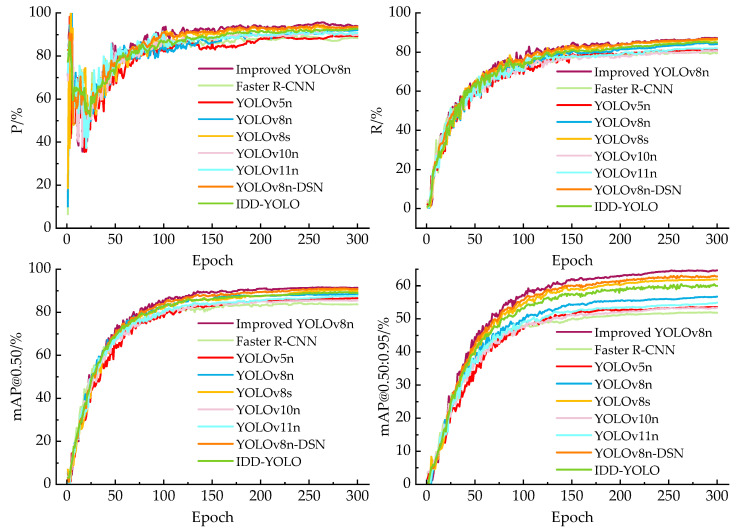
Training convergence curves of P, R, mAP@0.50 and mAP@0.50:0.95 for the improved YOLOv8n and the compared models.

Figure 11 shows training convergence curves of P, R, mAP@0.50 and mAP@0.50:0.95 on the validation set for the proposed model and the compared methods. P curves fluctuate noticeably at the early stage and become stable in the middle and late periods. Our proposed model is located above the curve group, indicating stronger suppression of false detections for defect targets under complex backgrounds. In the R curve, the proposed model maintains a higher recall level across most epochs, suggesting more complete defect detection with fewer missed cases. For the mAP@0.50 and mAP@0.50:0.95 curve, the proposed model not only has a higher upper limit of convergence, but also shows a more obvious advantage on mAP@0.50:0.95, reflecting improved bounding-box regression quality and fine localization capability at higher IoU thresholds.

**Figure 12:**
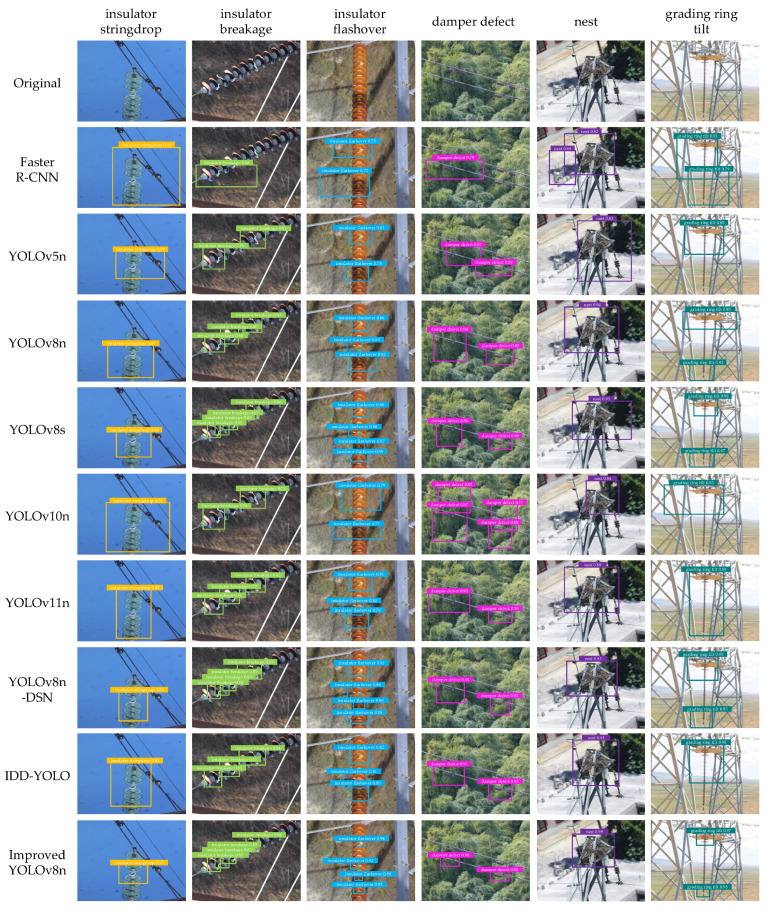
Detection results of the improved YOLOv8n and the compared models.

Figure 12 presents the detection results of the improved YOLOv8n and the compared models. In general, Faster R-CNN and some lightweight models are more prone to missed detections and false positives, and their predicted boxes show more noticeable localization offsets. For example, in damper defect and nest scenes with forest backgrounds, background textures and line structures interfere with each other, causing some models to produce redundant boxes or fail to fully cover the target regions. In contrast, our improved YOLOv8n delivers more stable performance across categories. It detects small defects more reliably, and its predicted boxes align more closely with object contours. Redundant boxes are reduced, and confidence scores are more consistent. In particular, for slender-structure targets such as insulator breakage and insulator flashover, as well as confusing cases such as grading ring tilt, the improved model more accurately localizes key regions and suppresses background interference, demonstrating stronger fine localization capability.

### 3.6. Visualization Analysis

**Figure 13:**
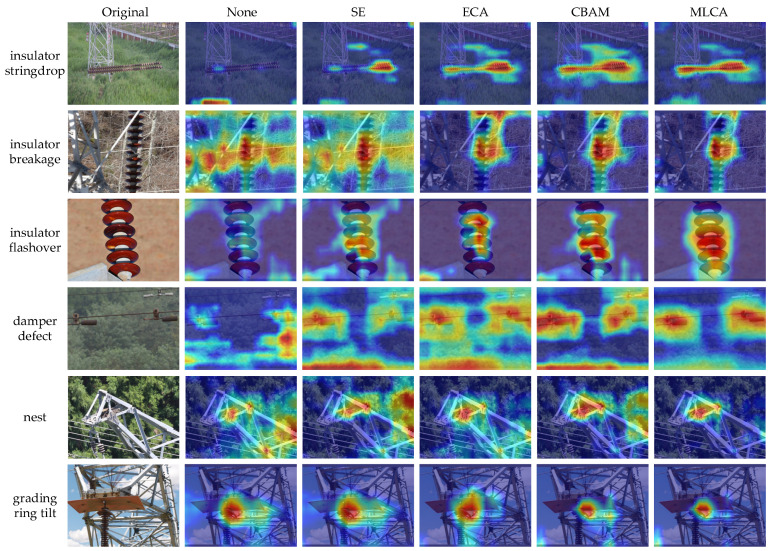
Heatmap comparison of different attention mechanisms.

To visually compare how different attention mechanisms focus on feature regions, we replace MLCA in the improved model with SE, ECA and CBAM, respectively. A model without any attention mechanism (None) is also included for reference. Figure 13 presents heatmaps for 6 defect categories under different attention settings.

As shown in Figure 13, the attention regions of None are relatively scattered and easily disturbed by complex backgrounds such as towers and vegetation. SE and ECA improve the focus on defect regions by optimizing the channel weight allocation, but their attention is still insufficiently concentrated in small-scale defect cases such as damper defect and grading ring tilt. CBAM combines channel and spatial attention and localizes defect regions more accurately. However, in highly cluttered scenes such as nest, part of the attention may still be assigned to non-target areas.

In contrast, MLCA shows more stable focusing behavior across defect categories. Its hot regions more concentratedly cover the defect core and key structures, while responses to surrounding backgrounds are more strongly suppressed. Heatmaps show that the target areas are brighter and the background areas are darker. These observations indicate that, by integrating local and global information and jointly modeling channel and spatial attention, MLCA helps to highlight the defect features in complex backgrounds and reduce irrelevant interference, thereby improving the stability and robustness of defect discrimination across scales.

### 3.7. Cross-Dataset Evaluation

To further verify the generalization ability of the proposed model, cross-dataset evaluation was conducted on the publicly available UPID [28] and SFID [6] datasets. Both datasets were constructed for power-insulator defect detection, where UPID corresponds to clear-weather scenes and SFID corresponds to foggy scenes. To ensure a fair comparison, the experimental settings on the public datasets were kept consistent with those used on our dataset, including the same experimental environment and hyperparameter configurations. The results of cross-dataset evaluation are presented in Table 7.

As shown in Table 7, on the UPID dataset, our improved algorithm achieves 97.1% mAP@0.50 and 82.9% mAP@0.50:0.95, both of which are the best among all compared methods. Compared with the baseline YOLOv8n, mAP@0.50 and mAP@0.50:0.95 are improved by 1.8% and 4.6%, respectively. Compared with YOLOv8n-DSN and IDD-YOLO, the proposed method still improves mAP@0.50 by 0.4% and 0.5%, and improves mAP@0.50:0.95 by 0.7% and 0.9%, respectively. On the SFID dataset, the proposed method also achieves excellent results, with 99.3% mAP@0.50 and 87.0% mAP@0.50:0.95. Since SFID is a foggy-scene dataset further constructed on the basis of UPID, these results indicate that the proposed method still maintains strong robustness under low-visibility conditions, complex backgrounds, and synthetic fog environments, while exhibiting more stable localization performance, especially under higher IoU thresholds.

## 4. Discussion

From the perspective of engineering application, the significance of the proposed method is not limited to improving the performance metrics of a single detection model, but also lies in its potential to be embedded into the UAV-based transmission and distribution line inspection workflow. Afanaseva and Tulyakov [29] pointed out that an information and control system for the condition monitoring of transmission and distribution lines is a multi-stage and systematic engineering process. It should include requirement analysis, architecture design, data acquisition, data processing and analysis, data visualization, as well as system testing and implementation, and it may adopt a modular architecture or service-oriented architecture to organize databases, communication systems, analytical algorithms, and visualization tools.

**Figure 14:**
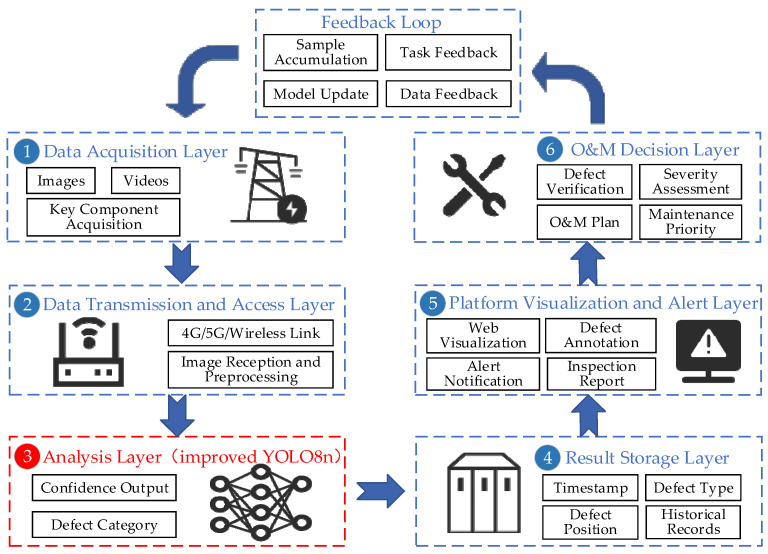
Application framework of the proposed method in a UAV-based power line monitoring system.

From this system perspective, the improved YOLOv8n defect detection algorithm proposed in this paper can be embedded into a UAV-based power line monitoring system, as shown in Figure 14:

Specifically, the UAV-based power line monitoring system consists of six connected stages:

(1) In the Data Acquisition Layer, UAVs equipped with visible-light imaging devices are used to inspect and collect images of the transmission and distribution lines and their key components.

(2) In the Data Transmission and Access Layer, the collected image data are transmitted through wireless communication links to edge-computing nodes or back-end servers, where data access and basic preprocessing are completed.

(3) In the Analysis Layer, the improved YOLOv8n model proposed in this paper is used to detect and classify defect targets in the images, and outputs information such as defect location, category, and confidence score.

(4) In the Result Storage Layer, the detection results are converted into structured information that can support operation and maintenance (O&M) management, such as defect category, confidence score, corresponding line section, tower number, equipment position, and inspection time, and are then written into the monitoring database in a unified format.

(5) In the Platform Visualization and Alert Layer, the information stored in the database can be visualized through web-based or mobile platforms, forming defect lists, inspection reports, annotated image results, and historical inspection records. The monitoring platform can not only provide defect alarms, but also link with historical inspection records and equipment archives to support maintenance personnel in formulating repair plans and reinspection strategies.

(6) In the O&M Decision Layer, O&M personnel use the collected data and the outputs displayed by the visualization interface to conduct defect verification, severity assessment, and maintenance-priority ranking, and then formulate scientific and reasonable maintenance plans.

The framework shown in Figure 14 also reflects a closed-loop O&M process. New inspection data and manual verification results accumulated on the platform can be fed back to the model training stage for sample supplementation, model updating, and algorithm optimization, thereby forming a closed-loop workflow. Such a closed-loop design is also more consistent with the practical needs of long-term operation and continuous optimization in transmission and distribution line monitoring systems.

In summary, Figure 14 illustrates the potential deployment framework of our proposed method in a UAV-based transmission and distribution line inspection system, namely, as an intelligent recognition and analysis module that works collaboratively with front-end perception, data transmission, platform visualization, and back-end decision-support modules. Therefore, the proposed work has potential applicability in real digital monitoring scenarios for transmission and distribution lines.

## 5. Conclusions

This paper proposes a lightweight power line defect detection model based on an improved YOLOv8n. Through case analysis, the following conclusions are obtained:

1. For the network structure, in the downsampling stage, the ADownPro module is designed to strengthen feature interaction while reducing computational cost. In the feature extraction stage, the CSPPC module is introduced to replace part of the C2F modules, reducing redundant computation and enhancing feature representation. In the detection head, the MLCA attention mechanism is embedded to combine channel-spatial and local-global information, thereby improving the focusing ability of defect regions under complex backgrounds.
2. For the optimization objective, the SAMQ-EIoU loss function is proposed. By integrating an iso-center scale transformation, scale-factor annealing, and a focal-style quality reweighting strategy, the training process gradually shifts from coarse alignment to fine localization, thereby strengthening bounding-box regression at high IoU thresholds.
3. Through comparison with existing methods, the proposed method achieves superior detection accuracy and model lightweightness. It attains 91.4% mAP@0.50 and 64.5% mAP@0.50:0.95, while reducing the parameters and FLOPs to 1.59 M and 4.9 G, respectively. In addition, the cross-dataset evaluation on the public UPID and SFID datasets further verify the robustness and generalization ability of the proposed method under different data distributions and weather conditions. These results indicate strong potential for UAV-based inspection of power transmission and distribution lines under limited computational resources.

It should be noted that the algorithm proposed in this paper has not yet been deployed and verified on real-edge devices, and further research will be conducted in this regard.

## Figures and Tables

**Table 1:** Experimental environment.

Item	Specification
Operating system	Windows 11
CPU	12th Gen Intel(R) Core(TM) i5-12490F
GPU	NVIDIA GeForce RTX 4060
Python	Python 3.8.20
CUDA	11.8

**Table 2:** Experimental hyperparameter settings.

Hyperparameter	Value
Input image resolution	640 × 640
Epoch	300
Batch size	32
Optimizer	SGD
Initial learning rate	0.01
Momentum	0.9

**Table 3:** Comparison of detection accuracy between improved YOLOv8n and YOLOv8n.

Model	AP@0.50/%						mAP@0.50/%	mAP@0.50:0.95/%
	Insulator Stringdrop	Insulator Breakage	Insulator Flashover	Damper Defect	Nest	Grading Ring Tilt		
YOLOv8n	83.7	74.9	82.5	97.9	99.1	90.5	88.3	56.6
Improved YOLOv8n (Ours)	83.9	78.2	89.2	98.1	99.3	99.5	91.4	64.5

**Table 4:** Results of the ablation experiment.

YOLOv8n	ADownPro	CSPPC	MLCA	SAMQ-EIoU	P /%	R /%	mAP@0.50/%	mAP@ 0.50:0.95/%	Params /M	FLOPs/G	FPS
√ ^1^					91.9	84.0	88.3	56.6	3.01	8.2	130.3
√	√				92.3	84.6	89.4	57.0	2.47	7.1	136.9
√	√	√			92.1	84.5	88.8	56.8	1.58	4.9	157.4
√	√	√	√		93.2	85.7	89.8	59.2	1.59	4.9	151.8
√	√	√	√	√	94.0	86.6	91.4	64.5	1.59	4.9	152.1

^1^ √ denotes that the corresponding module has been added.

**Table 5:** Results of the data augmentation ablation experiment.

None	M1	M2	M3	M4	M5	M6	mAP@0.50/%	mAP@0.50:0.95/%
√ *							87.3	59.8
√	√						87.8	60.4
√	√	√					88.9	61.8
√	√	√	√				90.4	62.5
√	√	√	√	√			90.2	62.4
√	√	√	√	√	√		91.1	63.9
√	√	√	√	√	√	√	91.4	64.5

* √ denotes that the corresponding module has been added.

**Table 6:** Results of comparative experiments.

Model	P/%	R/%	mAP@0.50/%	mAP@0.50:0.95/%	Params/M	FLOPs/G	FPS
Faster R-CNN	88.2	79.3	83.6	51.9	41.46	109.0	11.7
YOLOv5n	89.3	80.9	86.4	53.5	1.77	4.2	109.1
YOLOv8n	91.9	84.0	88.3	56.6	3.01	8.2	130.3
YOLOv8s	92.9	86.3	90.2	61.8	11.11	28.5	52.6
YOLOv10n	90.0	80.5	85.4	53.2	2.70	8.4	107.9
YOLOv11n	90.1	81.8	87.5	54.6	2.58	6.4	127.3
YOLOv8n-DSN	93.4	86.0	90.8	63.3	2.59	6.6	144.6
IDD-YOLO	93.1	85.1	90.6	59.7	2.85	5.1	113.2
Improved YOLOv8n (Ours)	94.0	86.6	91.4	64.5	1.59	4.9	152.1

**Table 7:** Results of cross-dataset evaluation.

Model	UPID		SFID	
	mAP@0.50/%	mAP@0.50:0.95/%	mAP@0.50/%	mAP@0.50:0.95/%
Faster R-CNN	92.5	75.6	98.3	80.0
YOLOv5n	94.2	76.5	98.7	80.9
YOLOv8n	95.3	78.3	99.0	82.6
YOLOv8s	96.4	81.3	99.2	85.5
YOLOv10n	93.6	76.4	98.6	80.7
YOLOv11n	94.8	77.2	98.9	81.5
YOLOv8n-DSN	96.7	82.2	99.4	86.3
IDD-YOLO	96.6	82.0	99.4	86.1
Improved YOLOv8n (Ours)	97.1	82.9	99.3	87.0

## Data Availability

The datasets used during the current study are available at https://github.com/tangtangdehaojiejie/Lightweight-Power-Line-Defect-Detection-Based-on-Improved-YOLOv8n/tree/main (accessed on 25 March 2026).

## References

[1] Chen J., Zhang X., Feng D., Li J., Zhu L. (2025). PLD-DETR: A Method for Defect Inspection of Power Transmission Lines. Electronics.

[2] Zhang J., Chen S., Wang W., Wang Q. (2025). Fully Autonomous Real-Time Defect Detection for Power Distribution Towers: A Small Target Defect Detection Method Based on YOLOv11n. Sensors.

[3] Wang X., Yang T., Zou Y. (2024). Enhancing Grid Reliability through Advanced Insulator Defect Identification. PLoS ONE.

[4] Hamam H. (2025). Rethinking Intelligence: From Human Cognition to Artificial Futures. Vokasi Unesa Bull. Eng. Technol. Appl. Sci..

[5] Xu Z., Tang X. (2025). Transmission Line Insulator Defect Detection Algorithm Based on MAP-YOLOv8. Sci. Rep..

[6] Zhang Z., Zhang B., Lan Z., Liu H., Li D., Pei L., Yu W. (2022). FINet: An Insulator Dataset and Detection Benchmark Based on Synthetic Fog and Improved YOLOv5. IEEE Trans. Instrum. Meas..

[7] Girshick R., Donahue J., Darrell T., Malik J. Rich Feature Hierarchies for Accurate Object Detection and Semantic Segmentation. Proceedings of the IEEE Computer Society Conference on Computer Vision and Pattern Recognition.

[8] Girshick R. Fast R-CNN. Proceedings of the IEEE International Conference on Computer Vision.

[9] Ren S., He K., Girshick R., Sun J. (2017). Faster R-CNN: Towards Real-Time Object Detection with Region Proposal Networks. IEEE Trans. Pattern Anal. Mach. Intell..

[10] Qiao S., Chen L., Yuille A. Detectors: Detecting Objects with Recursive Feature Pyramid and Switchable Atrous Convolution. Proceedings of the IEEE Computer Society Conference on Computer Vision and Pattern Recognition.

[11] Wang C., Yeh I., Liao H. Yolov9: Learning What You Want to Learn Using Programmable Gradient Information. Proceedings of the European Conference on Computer Vision.

[12] Wang A., Chen H., Liu L., Chen K., Lin Z., Han J., Ding G. (2024). Yolov10: Real-Time End-to-End Object Detection. Adv. Neural Inf. Process. Syst..

[13] Faisal M.A.A., Mecheter I., Qiblawey Y., Fernandez J.H., Chowdhury M.E.H., Kiranyaz S. (2025). Deep Learning in Automated Power Line Inspection: A Review. Appl. Energy.

[14] Yao G., Zhu S., Zhang L., Qi M. (2024). HP-YOLOv8: High-Precision Small Object Detection Algorithm for Remote Sensing Images. Sensors.

[15] Lou H., Duan X., Guo J., Liu H., Gu J., Bi L., Chen H. (2023). DC-YOLOv8: Small-Size Object Detection Algorithm Based on Camera Sensor. Electronics.

[16] Hong Y., Pan R., Su J., Li M. (2024). Infrared Image Detection of Defects in Lightweight Solar Panels Based on Improved MSRCR and YOLOv8n. Infrared Phys. Technol..

[17] Gao Y., Li Z., Wang Y., Zhu S. (2024). A Novel YOLOv5_ES Based on Lightweight Small Object Detection Head for PCB Surface Defect Detection. Sci. Rep..

[18] Tang Q., Su C., Tian Y., Zhao S., Yang K., Hao W., Feng X., Xie M. (2025). YOLO-SS: Optimizing YOLO for Enhanced Small Object Detection in Remote Sensing Imagery. J. Supercomput..

[19] Lu Y., Li D., Li D., Li X., Gao Q., Yu X. (2024). A Lightweight Insulator Defect Detection Model Based on Drone Images. Drones.

[20] Terven J., Córdova-Esparza D.M., Romero-González J.A. (2023). A Comprehensive Review of YOLO Architectures in Computer Vision: From YOLOv1 to YOLOv8 and YOLO-NAS. Mach. Learn. Knowl. Extr..

[21] Diao Z., Guo P., Zhang B., Zhang D., Yan J., He Z., Zhao S., Zhao C., Zhang J. (2023). Navigation Line Extraction Algorithm for Corn Spraying Robot Based on Improved YOLOv8s Network. Comput. Electron. Agric..

[22] Chollet F. Xception: Deep Learning with Depthwise Separable Convolutions. Proceedings of the IEEE Conference on Computer Vision and Pattern Recognition.

[23] Zhao B., Guo A., Ma R., Zhang Y., Gong J. (2024). YOLOv8s-CFB: A Lightweight Method for Real-Time Detection of Apple Fruits in Complex Environments. J. Real-Time Image Process..

[24] Wan D., Lu R., Shen S., Xu T., Lang X., Ren Z. (2023). Mixed Local Channel Attention for Object Detection. Eng. Appl. Artif. Intell..

[25] Zhang X., Zuo G. (2025). Small Target Detection in UAV View Based on Improved YOLOv8 Algorithm. Sci. Rep..

[26] Luo X., Cai Z., Shao B., Wang Y. (2024). Unified-IoU: For High-Quality Object Detection. arXiv.

[27] Liu S., Shao F., Chu W., Dai J., Zhang H. (2025). An Improved YOLOv8-Based Lightweight Attention Mechanism for Cross-Scale Feature Fusion. Remote Sens..

[28] Tian Y., Ahmad R.B., Abdullah N.A.B. (2025). Accurate and Efficient Insulator Maintenance: A DETR Algorithm for Drone Imagery. PLoS ONE.

[29] Afanaseva O.V., Tulyakov T.F. (2025). A Methodology to Develop an Information and Control System to Monitor the Technical State of Power Transmission Lines. Elektroteh. Vestn..

